# Assessing the Spatial Scale Effect of Anthropogenic Factors on Species Distribution

**DOI:** 10.1371/journal.pone.0067573

**Published:** 2013-06-18

**Authors:** Marco Mangiacotti, Stefano Scali, Roberto Sacchi, Lara Bassu, Valeria Nulchis, Claudia Corti

**Affiliations:** 1 Museo Civico di Storia Naturale di Milano, Milan, Italy; 2 Dipartimento di Scienze della terra e dell’Ambiente, Università di Pavia, Pavia, Italy; 3 Sezione Sardegna, Societas Herpetologica Italica, Oristano, Italy; 4 Sezione di Zoologia “La Specola”, Museo di Storia Naturale dell’Università di Firenze, Florence, Italy; Consiglio Nazionale delle Ricerche (CNR), Italy

## Abstract

Patch context is a way to describe the effect that the surroundings exert on a landscape patch. Despite anthropogenic context alteration may affect species distributions by reducing the accessibility to suitable patches, species distribution modelling have rarely accounted for its effects explicitly. We propose a general framework to statistically detect the occurrence and the extent of such a factor, by combining presence-only data, spatial distribution models and information-theoretic model selection procedures. After having established the spatial resolution of the analysis on the basis of the species characteristics, a measure of anthropogenic alteration that can be quantified at increasing distance from each patch has to be defined. Then the distribution of the species is modelled under competing hypotheses: H_0_, assumes that the distribution is uninfluenced by the anthropogenic variables; H_1_, assumes the effect of alteration at the species scale (resolution); and H_2_, H_3_ … H_n_ add the effect of context alteration at increasing radii. Models are compared using the Akaike Information Criterion to establish the best hypothesis, and consequently the occurrence (if any) and the spatial scale of the anthropogenic effect. As a study case we analysed the distribution data of two insular lizards (one endemic and one naturalised) using four alternative hypotheses: no alteration (H_0_), alteration at the species scale (H_1_), alteration at two context scales (H_2_ and H_3_). H_2_ and H_3_ performed better than H_0_ and H_1_, highlighting the importance of context alteration. H_2_ performed better than H_3_, setting the spatial scale of the context at 1 km. The two species respond differently to context alteration, the introduced lizard being more tolerant than the endemic one. The proposed approach supplies reliably and interpretable results, uses easily available data on species distribution, and allows the assessing of the spatial scale at which human disturbance produces the heaviest effects.

## Introduction

Human presence and activities inevitably produce changes in the surrounding environment that may affect the occurrence of a species in a given territory: some species may go locally extinct because they cannot find suitable conditions, while some others may spread to new territories because of the new conditions. Consequently, anthropogenic habitat alteration is considered as one of the dominant forces shaping the spatial distribution of species [1,2], with outcomes that are sometimes beneficial for the species and sometimes not. The conservation biology has grown and evolved in response to human threats to the natural world in order to set the best scientific guidance to preserve sites and species of special interest. One of the central topics for conservation biologists is to be able to reliably evaluate how the distribution of a species is affected by anthropogenic habitat alteration and loss. The issue is crucial and may be the starting point to elaborate really effective conservation plans.

**Figure 1:**
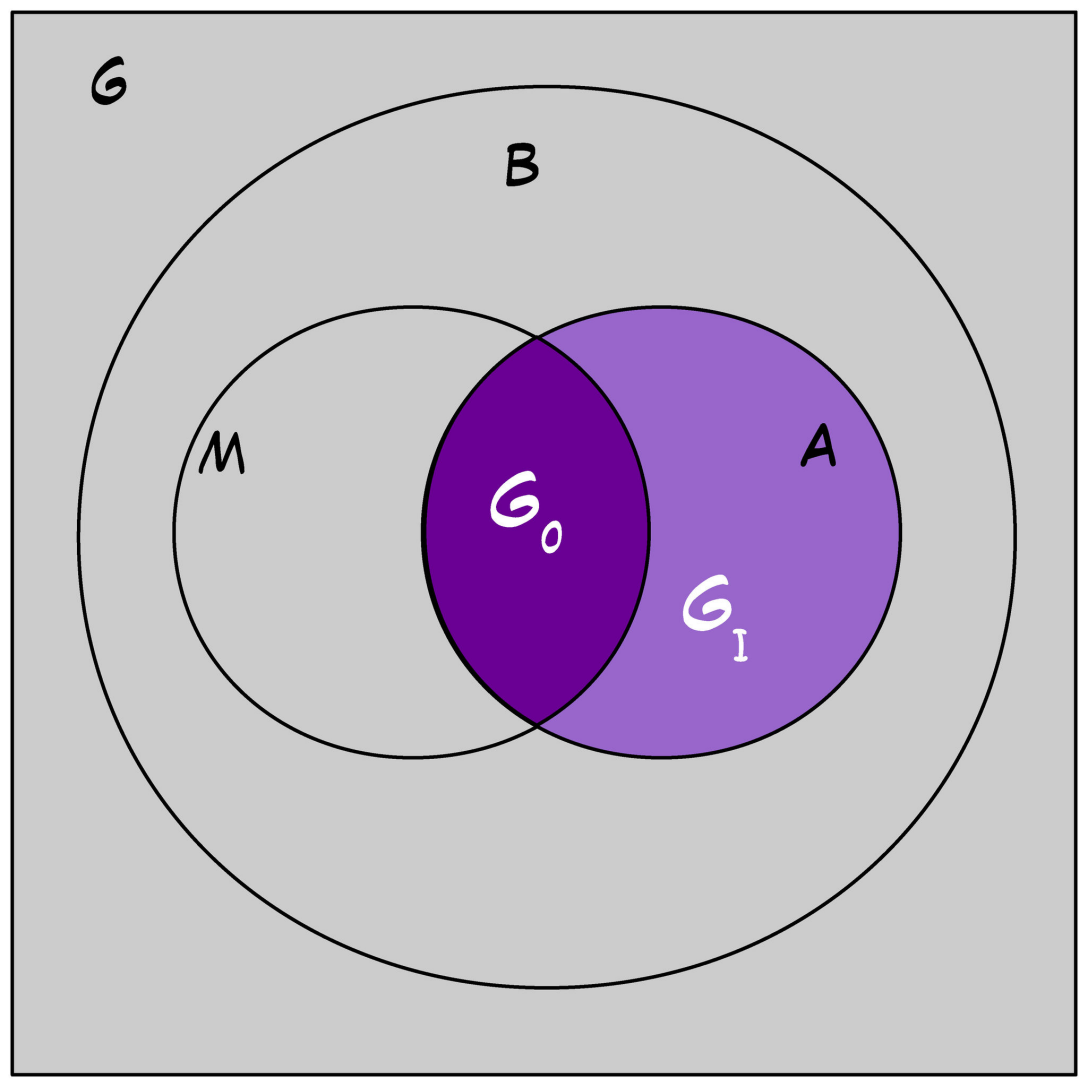
BAM diagram. Simplified version of the Biotic-Abiotic-Movement diagram from Sauge et al. [16]. “G” represents the geographic space. “B” is the part of G that presents the correct set of biotic conditions: in this simplified version B is assumed not to constrain species distribution [3,16]. “A” is the part of G that holds suitable abiotic conditions. “M” represents the sub-area of G that has been accessible and explored by the species. The intersection between M and A defines the species distribution (G_0_). G_I_ is the area that is potentially suitable, but has not been accessible to the species.

In the last two decades the development of techniques which allow modelling the spatial distribution of a species on the basis of the relationship between species and environment has given a powerful tool to face the problem: an increasing number of studies have actually made use of spatial distribution models (SDM) – also known as ecological niche models [3] – to answer questions related to conservation tasks [4–6]. As their use has increased, greater and greater attention has been paid to the effects of the spatial scale (with different meanings) on the modelling process [7–14]. Besides the well-known importance of spatial scale in ecology [15,16], this particular attention comes from the plain consideration that the distribution of a species is the result of the combination of different factors (summarized in the BAM diagram – Figure 1 – where “B” stands for biotic interactions, “A” for abiotic conditions, and “M” for movement, i.e. area accessibility [3,17]), which act in the geographic space and are related each other in a scale-dependent way [11,18]. The majority of the studies focusing on spatial scale were devoted at analysing spatial grain and/or extent (sensu Wiens [15]) and how SDM respond to their changes [7,8,10,12,14]. These two components in the SDM studies have been generally associated respectively to the resolution (pixel size) and the geographic range (extent of the study area) at which the models are developed [8]. A relatively less studied issue, which is related to the “extent” side of spatial scale [15,19], was the relationship between the characteristics of the surroundings of a pixel on the predicted suitability of that pixel [9,15]. This aspect of extent (known as “patch context” in landscape ecology [19]) may play an important role in shaping the distribution of a species, for example by conditioning the dispersal ability [20–22], i.e. the “M” factor of the BAM diagram. A support for the relevant role of patch context comes from the few studies that explicitly incorporate it among the variables employed to build the models [9,13,23]: all of them found a significant effect of patch context, and in some cases [9] they were able to assess the spatial extent to be considered “context”. Nevertheless, the experimental design adopted by all these studies was based on a sample of intensively surveyed sites, thus limiting the geographic range of the study area and preventing the assessment of general pattern of dependence between the species distribution and the spatial scale of the context. Furthermore, context was defined without an explicit reference to the anthropogenic alteration, thus none of the above studies explicitly addressed the link between human-induced alteration and species occurrence, nor the scale at which human activities act on species distribution. In the light of this lacks, our aim is to propose a generalizable approach to assess the occurrence and the spatial scale (in term of context extent) of the anthropogenic effect on the species distribution by combining species presence data, SDM and information-theoretic model comparison (IMTC) [24].

SDM have been developed to predict the distribution of a species on the basis of known occurrences and a set of environmental layers (e.g., annual precipitation, annual mean temperature, land use): the model produces suitability scores for all the sites within a given area by capturing species-environment relationship [5]. Since links between species and environment are the key-step of the procedure, these models are also known as ecological niche models [3,6]. Without entering the debate about nomenclature and related concepts [3,25–27], we adopt the term “SDM” according to Peterson and Soberón [3], since the objective of our modelling is the estimate of the actual distribution, taking into account accessibility (though indirectly; see below) [3]. Depending on the biological questions, SDM may be used as a predictive or explanatory tool: while in the former case SDM are employed to make predictions to new sites (either in space or in time) [28,29], in the latter case SDM are built to investigate the causes of the observed distribution, i.e., models serve to evaluate the effects of the variables used to build the SDM on the performance of the model itself: it is implicitly assumed that any effect on the model corresponds to a relationship between the variable and the distribution [30,31]. Our use of SDM belongs to this latter case. ITMC is a general well known approach in ecology and other branches of sciences [32]. It allows comparing different hypotheses, each represented by a model, searching for the best one [33]. The competing models are ranked on the basis of an information criterion (such as the Akaike Information Criterion or the Bayesian Information Criterion) which measures the amount of the information not captured by the model, weighed for the complexity of the model itself [32,33]. In addition to the ranking of the models from the best to the worst, it is also possible to evaluate how much a model, and its underlying causal hypothesis, is better than another one [33]. Our idea is to combine these two techniques in order to answer the initial question - how is the observed distribution of a species affected by anthropogenic habitat alteration? - paying attention to the spatial scale of the processes involved.

## Methodological Scheme

The approach can be summarized as follows: i) establishing the resolution (grain component of the spatial scale) of the analysis, taking into consideration the characteristic of the species under study; ii) defining a measure of habitat alteration that can be applied at different spatial scales (i.e. quantifiable at increasing distance from each pixel); iii) obtaining a set of geographic layers each representing the alteration at a given scale iv) formulating a set of competing hypotheses with or without the habitat alteration factors; v) translating each hypothesis into an SDM; vi) comparing the obtained SDM using the ITMC, in order to assess the SDM (and the hypothesis underlying it) best explaining the species occurrence. In formulating the competing hypotheses, the null hypothesis assumes that anthropogenic changes do not affect distribution (H_0_): an approximation of H_0_ is a model which includes only climatic and topographical variables (that is to say a topoclimatic model), since it predicts species occurrence when habitat variables reflect their climatic potential and all the potentially suitable areas are accessible to the species [3]. Even if these assumptions may seem unrealistic, they serve as a starting point for comparing the models generated under hypotheses that call for anthropogenic effects. We are aware that other non-anthropogenic factors may actually affect both dispersal and pixel characteristics and consequently trim the potential distribution, but we consider that: i) a careful and expert-based choice of the extent of the study area may help producing a more realistic H_0_ [6,34]; ii) the aim is not to build the fittest SDM, but to assess if adding anthropogenic factors improves the fit of the model. We expect that anthropogenic alteration acts either on the quality of a pixel as on the permeability of its surroundings, thus reducing or modifying the potential distribution of a certain amount [35]. So we are interested in the trimming effect produced by an anthropogenic variable on the topoclimatic model.

Within the previous framework, each of the alternative HYPOTHESIS H_1,_ H_2_ etc. should include the topoclimatic variables and one or more layers that describe anthropogenic alterations at different scales. In this way a set of nested models are obtained, each differing from H_0_ only for the presence of the human effect among variables. In order to illustrate our theoretical scheme at work and explain the detail of the procedure, we applied it to a set of data on two Sardinian lizard species.

### Species and study area

We applied our scheme to the case of two congeneric lacertid lizards which inhabit a large Mediterranean island. The choice of a large island is motivated by two reasons: i) to bypass the problem of defining the limits of the study area; ii) to avoid the problem of resources limitation due to small dimension of the area.

Sardinia (40.10° N, 8.95° E, Italy) is a large island (24090 km^2^) located in the Tyrrhenian Sea (Western Mediterranean Sea). The numerous islets that surround the main island were not included, because of the particular conditions they represent. Following the Köppen-Geiger climate classification [36], the climate belongs to the Cs type (Warm temperate climate with dry summer), subtype a (hot summer) and b (warm summer). Vegetation consists of forests (primarily evergreen oak and cork trees and deciduous woods with oak and chestnut) and Mediterranean scrubs [37].

The island is inhabited by both endemic and introduced species [38], including the lacertid lizards of the genus *Podarcis*, the Tyrrhenian wall lizard *P* *. tiliguerta* (Gmelin, 1798), and the Italian wall lizard *P* *. siculus* (Rafinesque-Schmaltz, 1810). The former is a small lizard (snout-ventral length in general up to 6.5 cm [39,40]) endemic to Corsica and Sardinia and their surrounding islets. It shows a great ecological plasticity occurring preferentially in sparse scrub with rocky outcrops, but avoiding patches with too dense woodland and habitats characterized by intensive agriculture [40,41]. It ranges from the sea level up to about 1800 m a.s.l. [41,42]. The Italian wall lizard, is a medium-sized lizard, slightly larger than *P* *. tiliguerta* (snout-ventral length up to 9 cm [39]), occurring in southern-central Europe (continental Italy and on many islands, coastal region of Slovenia and Croatia and some areas of Montenegro). It shows a great colonization ability (naturalized populations are found in Portugal, Spain, France, Turkey, Tunisia, Libya and USA [39,43]), and it has been introduced in Sardinia in subsequent historical or protohistorical times, maybe following commercial routes from Sicily [44,45]. The Italian wall lizard can use many different habitats (including anthropic ones) characterized by sunny and open areas [39,41–43]. These two species have been chosen because of their different response to anthropization, the Italian wall lizard being more tolerant. So we should expect differences in the effects of anthropogenic habitat alterations between them.

A total of 934 species occurrence points were collected during surveys from 2000 and 2009 as part of the study carried out by the Museo di Storia Naturale dell’ Università di Firenze, Sezione di Zoologia and the Sezione Sardegna of the Italian Society of Herpetology S.H.I., (*Societas Herpetologica Italica*) in order to produce the Italian Herpetological Atlas and Fauna d’Italia Reptilia volume [38,43] and by the Regione Autonoma della Sardegna for the realisation of the regional atlas (still on-going). This research was carried out under permits released by the Italian Ministry of Environment. None of the animals was captured and/or manipulated, and species identification was made by sight (this kind of study does not need permits under the current Italian and European legislation; furthermore, the sampled area falls outside any kind of restricted areas which needs permission). Geographical locations of species occurrence points were recorded using a GPS. No data about absence were available, so the sample represented a presence-only dataset. Data were resampled to a 100 × 100 m grid obtaining respectively 291 presence cells for *P* *. tiliguerta* and 355 for *P* *. siculus* . We chose this spatial grain on the basis of available data about the dispersal ability of the lizards of the genus *Podarcis*. In particular, maximum home range dimension has been demonstrated to be of about 300 m^2^ [46–48] and homing ability has been registered up to 150 m distance [46,49]. So, a 100 m wide cell is expected to enclose the whole home range of a lizard and to represent its dispersal ability; for these reasons this scale can reliably represent the species spatial scale.

Two kinds of environmental data at the same spatial resolution (100 m) were directly obtained from the web: a digital elevation model (DEM) (http://srtm.csi.cgiar.org) and a map of the land use relating to 2008 (http://www.sardegnageoportale.it). Starting from this cartographic base, two sets of variables were generated: i) topographic, from DEM (altitude, slope and potential solar radiation), and ii) anthropic, from land use (see below). We had initially chosen not to use the many bioclimatic databases available on the web because none of them is detailed enough to allow 100 × 100 m resolution (e.g., the 19 bioclimatic variables of the Worldclim series, www.worldclim.org, has a maximum resolution of about 1 × 1 km at the latitude of Sardinia). Nevertheless, we compared the variables derived from DEM (resampled to 1 km) to those from Worldclim [50]: the high values of the correlation coefficients among most of them (12 out of 19; Table S1) supported the choice of using altitude, slope and potential solar radiation as surrogates of these bioclimatic variables. On the other hand, since some ecologically meaningful climatic variables were not well represented in our H_0_, the null model would have been easily improvable, via correlation, by any kind of appended variable. To avoid this issue, we decided to include also those bioclimatic variables that were not highly correlated with the topographic ones or among each other (i.e., bio2, mean diurnal temperature range; bio7, temperature annual range; bio13, precipitation of wettest month; bio15, precipitation seasonality). All these extra factors were previously interpolated to 100 m spatial resolution. Then, the final set of topoclimatic variables was composed by three topographic and four climatic layers.

Anthropic variables were defined taking into consideration the two sides of the spatial scale: grain (resolution) and extent (patch context). The variable “alteration” was built to quantify the direct habitat loss following anthropogenic alteration (sensu Fahrig [35]), and was modelled at the species scale by coding each cell with zero (not altered) or one (altered). Considering the historical background of land use in Sardinia, habitats completely not influenced by human activities are very scarce. Thus we defined “alteration” an intensive and direct exploitation of land, currently on-going and such that it prevents semi-natural structure from evolving (from this perspective, cropland and urban settlements are examples of altered habitat; cork-oak forest and pastures have to be considered not altered). We reclassified the land-use categories on the basis of this definition and the peculiar characteristic of Sardinian territory (table S2). To quantify the alteration of the context of each pixel at different spatial scales, we first chose a set of increasing distances, starting from the one that allow defining as “context” the eight pixels directly in contact with the considered one (150 m, 500 m, 1 km, 5 km, 10 km, 15 km, 20 km). For each distance we obtained the proportion of altered pixels (defined by variable “alteration”) within a circle centred in the centre of the pixel and with a radius equal to the distance considered. Then we computed the spatial correlation among all these “context alteration” layers (table S2) and we selected: i) the minimum distance at which the correlation coefficient (r_s_) with “alteration” did not exceed 0.70; ii) the subsequent minimum distances at which correlations with the previous selected layer fell below 0.70. In this way two distance ranges were chosen: 1 km (the finest scale for pixel context – variable PC-fine) and 15 km (variable PC-coarse). In conclusion, we derived three anthropic variables: “alteration” which represents the pixel state with respect to human disturbance; PC-fine and PC-coarse which measures context alteration at two different spatial scales. The spatial correlations among the final set of variables did not show coefficients exceeding 0.70 (maximum value was 0.68; table S3). Thus, we were able to build SDM under four causal hypotheses: H_0_ (topoclimatic), H_1_ (topoclimatic and alteration), H_2_ (topoclimatic, alteration and PC-fine) and H_3_ (topoclimatic, alteration and PC-coarse).

### Modelling procedures

Many algorithms are available to model the spatial distribution of a species [6]. Among them, we chose the maximum entropy method [51] implemented by the software Maxent (version 3.3.3e; http://www.cs.princeton.edu/~schapire/maxent/), because it has been demonstrated to outperform other methods [52], and does not require absence data (it uses presence sites and background sites) [51]. When using Maxent, two fundamental decisions have to be taken. The first deals with the choice of the background. Besides the set of the presence points, indeed, Maxent uses a set of background points, which allow characterizing the environment available to the species. The choice of the background is particularly critical because it may influence the results if the sampling effort across the study area is not homogeneous [53]. A general framework to deal with sampling bias is getting the background points with the same bias as the presence sample [54]. Our sampled areas were not homogeneously distributed across the Sardinian island, but they concentrated along the road network (Figure S1). So, we decided to take the background points from a 2 km wide buffer along the roads (the map of the road network was obtained from http://www.sardegnageoportale.it). The selected distance represented the mean plus one standard deviation of the distances between each presence point and the nearest road. The background points were then generated by extracting 10,000 random points from this reduced area and adding the presence points. In this way almost the same sample bias affects presence sites and background points [54]. The second decision concerns the kind of “features” which have to be included in the model. Maxent, in fact, does not use the raw variables, but their transformed versions (called “features”) [51]. Maxent handles five kinds of features: linear, quadratic, product, threshold and hinge. The use of features instead of the raw variables is preferable when a complex process has to be modelled [55], but it leads to some over-fitting problems [55,56]. To avoid this theoretical risk we developed all the models by using only the hinge features, reducing redundancy [55].

### Models evaluation and comparison

Model discriminating performance was estimated using AUC (Area Under the receiver operating characteristics Curve), which is a threshold-independent measure of the ability of the model to discriminate between background and presence sites [48]. AUC is widely applied in species distribution modelling to assess the classification ability of a model, but its employment in presence/background studies has been criticised by some authors [57–59] because: i) “AUC assumes nothing about the relative costs of errors of omission and commission” [58]; ii) high AUC values may not reflect real accuracy if the “test presences disproportionally represent inhabited areas” [59]. In order to partially overcome these limits, we adopted two strategies: firstly, as suggested by Smith [59], we tested the significance of the observed AUC through randomization [60]. For both the species we randomly extracted from the background 999 samples of the same size as the observed ones ( *P* *. tiliguerta* , N = 291; *P* *. siculus* , N = 355); we used these pseudoreplicates to generate 999 models and obtain the distribution of the AUC values. Then, we computed the probability associated to the observed AUC using the formula:

(eqn. 1) $$\text{P}=\frac{\text{v}+\text{1}}{\text{n}+\text{1}}$$

where *v* is the number of values in the AUC distribution that exceed the observed one, and *n* is the total number of the pseudoreplicates [61]. Secondly, we converted the continuous suitability scores of Maxent output into binary outcomes (0 for absence, 1 for presence) and then we measured sensitivity (frequency of presence sites correctly classified) and specificity (frequency of pseudo-absences correctly classified) of each model. The cut-off value needed for the output conversion was chosen in order to maximize the two measures, i.e. to obtain equal sensitivity and specificity [62].

Model selection was carried out by means of IMTC [24]. We used the small sample unbiased Akaike Information Criterion (AICc) [63], since all the tested models had a number of free parameters that exceeded *n*/40 (*n* is the number of the observations; table 1) [32,33]. The models were ranked on the basis of the difference between the model AICc and the minimum value of AICc among the models. Also the Akaike weights (w_i_) were computed [33]. Akaike weights can be interpreted as the probability that model *i* is the best model for the observed data, given the candidate set of models [32]. The AICc were calculated with ENMtools ver. 1.3 (http://enmtools.blogspot.com/) [56,64].

**Table 1:** Models performance and comparison.

Species			Hyp.		k	AUC		Thres.	Sens. = Spec.			AICc		Δi
*P. siculus*			H_2_		45	0.774		0.394	0.692			6261.680		0.000
			H_3_		46	0.750		0.391	0.657			6314.613		52.933
			H_0_		41	0.745		0.398	0.665			6319.105		57.425
			H_1_		44	0.747		0.395	0.660			6319.512		57.832
*P. tiliguerta*			H_2_		56	0.811		0.425	0.731			5117.208		0.000
			H_3_		54	0.774		0.453	0.703			5194.409		77.201
			H_1_		45	0.768		0.465	0.700			5202.807		85.599
			>H_0_		45	0.747		0.460	0.678			5250.413		133.205

Hyp: hypothesis used to build the model (H_0_, topoclimatic; H_1_, topoclimatic + alteration; H_2_, topoclimatic + alteration + PC-fine; H_3_ topoclimatic + alteration + PC-coarse); k number of estimated parameters in the model. AUC: Area Under the ROC curve: probability values obtained by randomization tests are lower than 0.001 for all the models. Thres: threshold, cut-off value for binary conversion of the original continuous output. Sens. = Spec.: sensitivity and specificity; the two measures are the same because the threshold was chosen to maximize both of them. AICc: Akaike Information Criterion corrected for small sample size. Δi: AICc difference between model i and the best model. Akaike weights are not presented since they were one for the best model in both species.

Finally, in order to assess the effect size of the three hypothesized factors (topoclimatic, pixel and context alteration), we applied a variation partitioning analysis [65] adapted to univariate model output, following Muñoz et al. [66]: the approach allows disentangling the pure effect of each factor while controlling for between-factors overlap. To evaluate the direction of the effect, we visually investigated marginal and single response curves generated by Maxent: the former plots the change in model prediction as each variable is varied, keeping all the other ones at their average sample value; the latter is obtained by constructing a model using only one variable at a time, and then plotting the response with respect to the possible values of the variable itself [51]. The combination of the two ways allowed evaluating the effect of the among-variables correlations on the response. To obtain confidence intervals of the response curves, we used the cross-validation procedure of Maxent, splitting the original sample in ten sub-samples.

## Results

**Figure 2:**
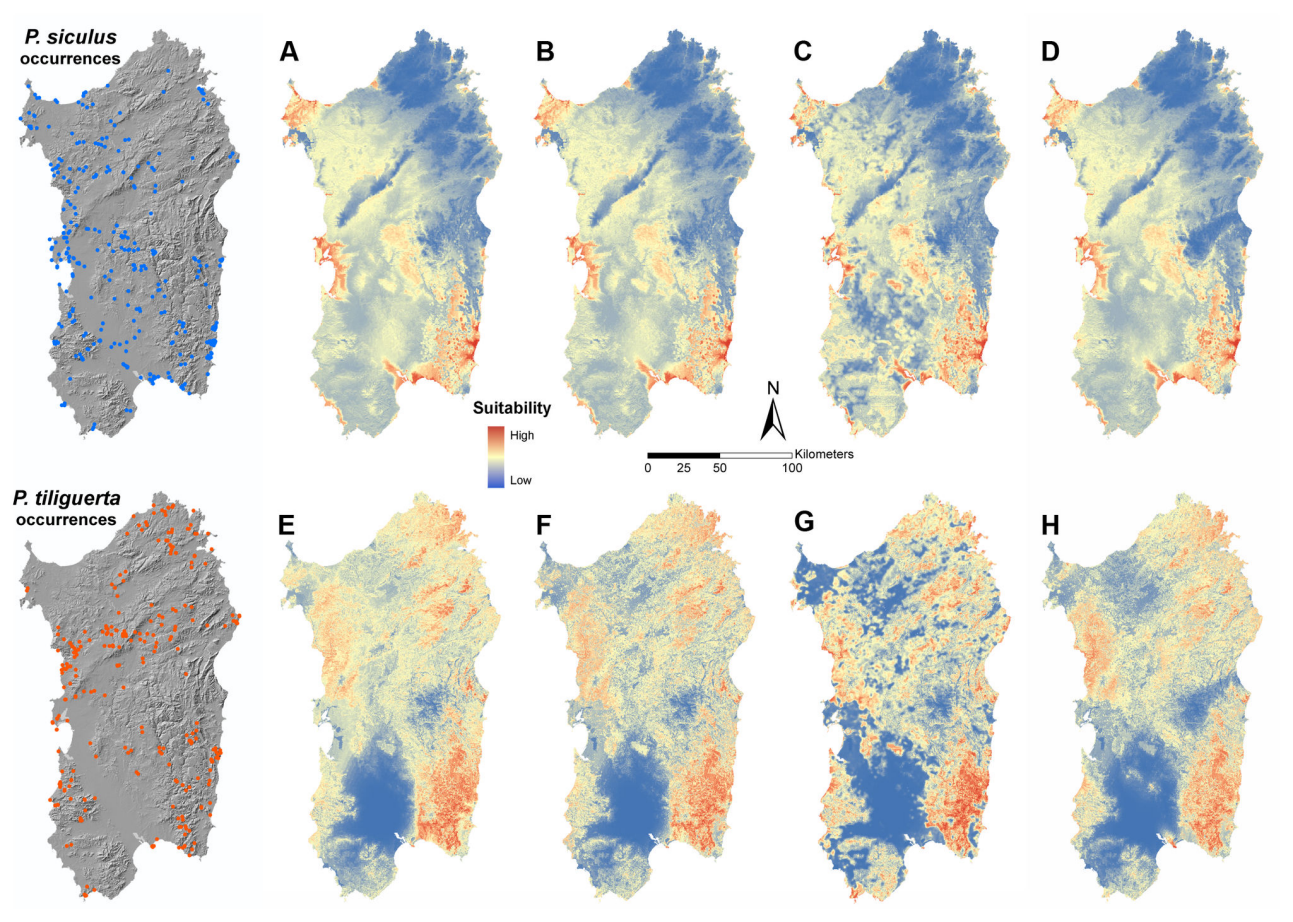
Maps of the competing SDM. A, B, C, D represent the models under hypotheses respectively H_0_, H_1_, H_2_ and H_3_ for *P* *. siculus* . E, F, G, H represent the same hypotheses for *P* *. tiliguerta* . Occurrences are also shown in separate maps.

A summary of the results concerning the developed SDM is reported in table 1. Looking at the discriminating performances (AUC), all the models show a significant deviation from chance and the best models for both species correspond to H_2_. Topoclimatic model (H_0_) is clearly the worst among those of *P* *. tiliguerta* , while it performs like H_1_ and H_3_ for *P* *. siculus* . The analysis of sensitivity and specificity values (table 1) leads to the same conclusion. The visual comparison of model maps (Figure 2) highlights that those built with PC-fine (H_2_) tend to better identify the unsuitable areas (blue colour), especially for the Tyrrhenian wall lizard. In both species, H_2_ hypothesis is also the most informative, showing the lowest AICc and having Akaike weight very close to one. The ranking of the models is slightly different between the two species (H_2_>H_3_>H_1_>H_0_ for *P* *. tiliguerta* ; H_2_>H_3_>H_0_≥H_1_ for *P* *. siculus* ), but all the models that have incorporated patch context characterizations are more informative than those that have simply used the alteration state of the pixels (H_1_).

**Figure 3:**
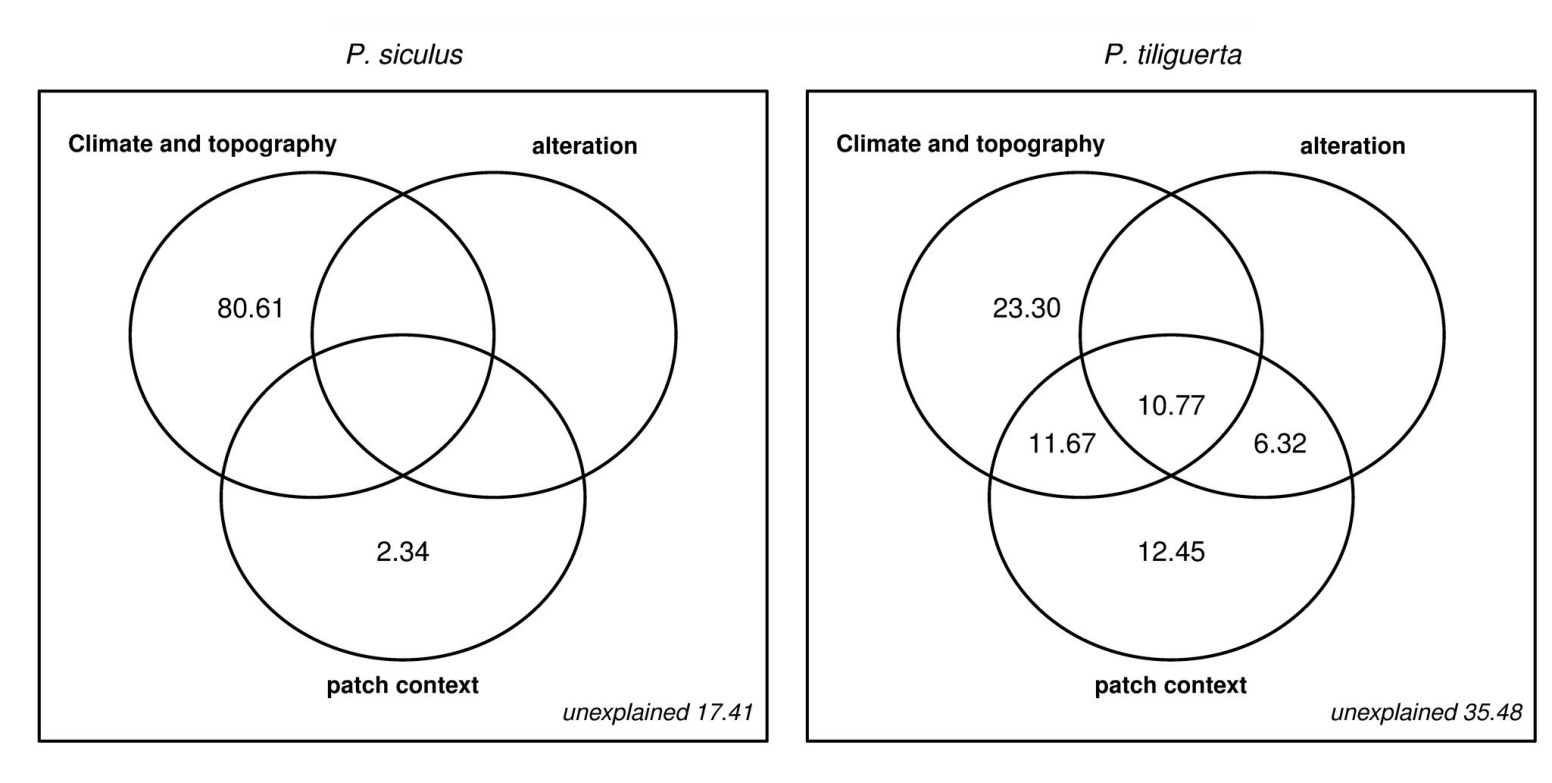
Variation partitioning diagrams for the H_2_ hypothesis for the two species. Circles represent variation explained by each factor (climate and topography, alteration, patch context). Numbers correspond to the percentage of variation associated to each circle subpart (pure, two-factor intersection, three factors intersection). The percentage associated to intersecting areas has not to be interpreted as interaction, but as a variation indifferently assignable to one or more factors [65]. Values smaller than 0.01% are not shown.

The variation partitioning of the output from H_2_ gives different results in the two lizards’ cases (Figure 3). The pure effect of climate and topography explains the largest part of model variation for *P* *. siculus* (80.61%), while the role of patch context is marginal (2.34%). Alteration shows nor a pure nor an overlapped effect on distribution and all the intersections are null. Results for *P* *. tiliguerta* are more complex: pure effects are found for climate and patch context (23.30% and 12.45% respectively) but not for alteration which shows complete overlap with patch context alone on one hand (6.32%), and with the other two factors together on the other hand (10.77%); topoclimatic and patch context factors share 11.67% of variation, after considering alteration.

**Figure 4:**
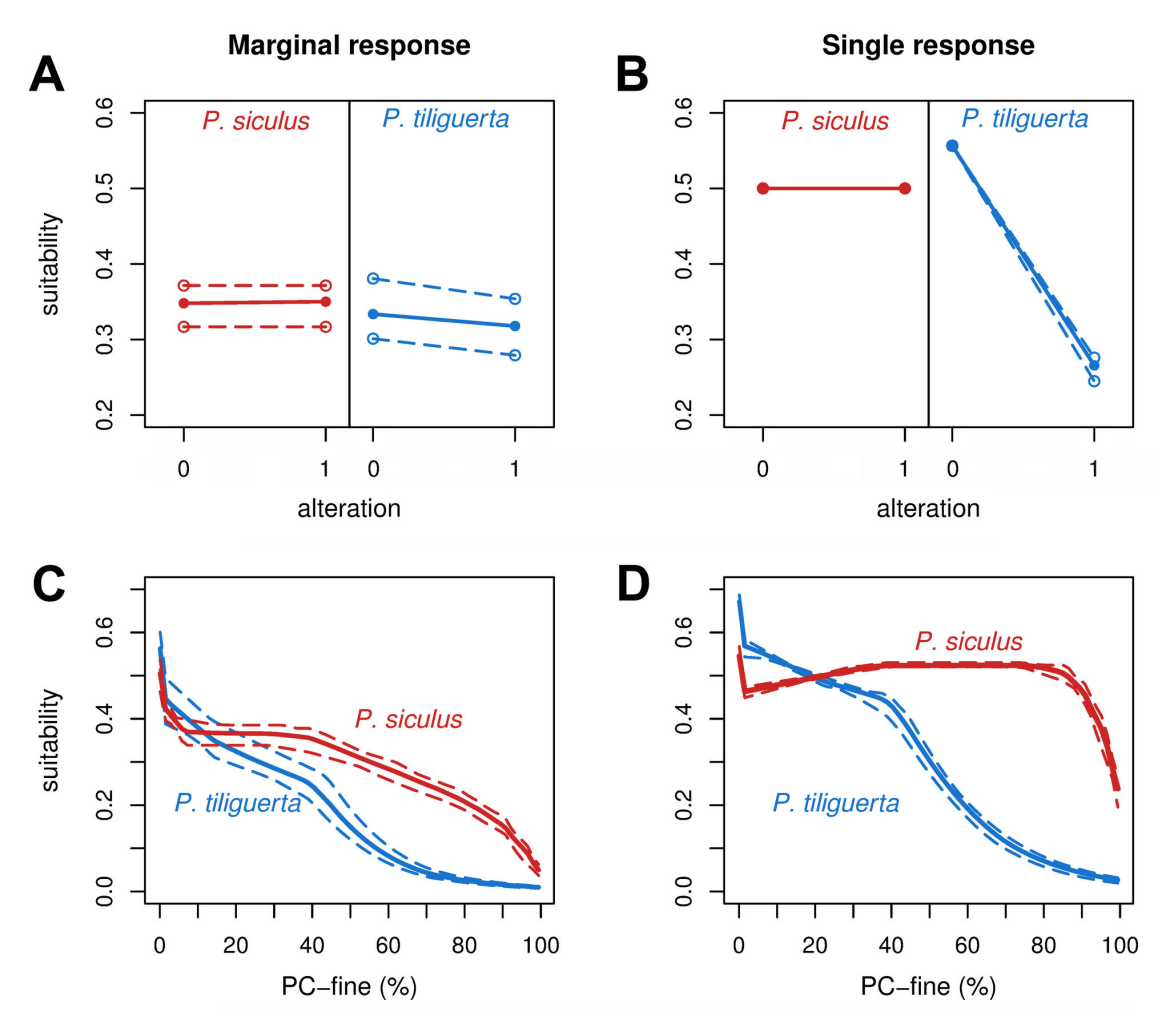
Response curve for the anthropogenic variable of the best model (H_2_). A: marginal response curves for the variable “alteration” (suitability is obtained from the full model keeping constant all the variables but alteration). B: single variable response curves for the same variable (suitability is obtained from a model including only the variable “alteration”). C: marginal response curves for PC-fine (patch context alteration). D: single variable response curves for the same variable. Solid lines indicate the mean of ten cross validated models; dashed lines represent the minimum and maximum range of the response curves.

The effects of the anthropogenic variables on the modelled suitability are graphically synthesized in Figure 4. All the response curves showed very narrow ranges, meaning low variation in response with the subsample considered. Marginal and single responses to habitat alteration show similar patterns: a negative relationship with suitability for the Tyrrhenian wall lizard, weak in the marginal response (Figure 4A), more evident in the single response (Figure 4B); no apparent relationship for the Italian wall lizard (Figure 4A, 4B). Also the responses to patch context (PC-fine) are quite different between the two species (Figure 4C, 4D): the Italian wall lizard seems to tolerate higher levels of altered context than the congeneric species, and suitability starts to decrease when alteration is above 40% (marginal response) or 85% (single response). On the contrary, *P* *. tiliguerta* appears to be more sensitive to altered context, since, in both graphs, suitability decreases with increasing alteration: the reduction becomes faster when alteration is above 40% (Figure 4C, 4D).

## Discussion

During recent years, scale dependent effects are attracting more and more interests of the studies on species distribution modelling [7–14]. There is increasing evidence that reliability of SDM as well as our understanding of the consequences of human-induced alterations largely depend on the scale at which they are modelled. This may have relevant effects on the conservation measures that can be implemented to preserve species. We proposed a generalizable two-steps approach to study the effect of the anthropogenic alteration of the patch context [19] on the spatial distribution of a species, in order to define up to what extent a distance can be regarded as “context” for a given species in a given geographical region. In our exemplification, based on the occurrence data of two lacertid lizards, the models built with the inclusion of variables accounting for habitat alteration of the context (PC-fine, PC-coarse) have proven to perform better than the basal models (table 1), and, more interestingly, than the models which incorporate only local alteration (H_1_). For both species, the best results were obtained setting the extent of patch context to 1 km (PC-fine, H_2_; table 1), and this distance has produced the strongest trimming effect on the potential distribution (Figure 2). PC-fine may be seen as a measure of isolation of each cell [35,67], a property strictly related to accessibility, the “M" component of the BAM diagram [17] [18],: the permeability of the context towards movements decreases as isolation increases, thus preventing to access the suitable patches [3] and negatively interfering with meta-population dynamics [68]. Clearly, only a variable that works at the context level of the spatial scale is able to collect information about patch isolation and consequently its use in model building is expected to produce more realistic SDM. Now the question becomes: how far do we have to go by the pixel? Our results show that an increase in the extent of the context from 1 to 15 km produces less informative model, thus indicating that a 15 km range is too large. The outcome seems plausible considering that M-factor does not depend only on the permeability of the context, but also on the dispersal ability of the species. In the case study this ability was about 100 m [46–49], so the extent of the context is not expected to become too large before a reduction in its effect on shaping the species distribution may occur. Previous studies about the influence of patch context on animals with relative small dispersal ability (but which made use of data from well surveyed sites and of other inference techniques), have set the effect at the scale of some kilometres (reptile and amphibian: 2 km [69]; beetles: till 2 km [19]; butterflies: from 1 km to 4 km [9]).

An additional relevant issue of this study is that the alteration of the pixel context has larger consequences than the alteration of the pixel itself (Figure 3). Also this result can be interpreted in the light of the study species and the definition of anthropogenic alteration we employed. Actually, both lizards (even though differences occur) tolerate human proximity, and may inhabit some kinds of anthropogenic habitats [40,43]. In this case suitability depends on the “semi-natural” microhabitats (e.g., gardens, rows of trees, ecotones between crops, wooded micro-patches) available inside each 100 × 100 m cell. The degree of suitability guaranteed by the occurrence of these micro-habitats depends on their amount and quality in addition to the species considered (e.g., the endemic lizard is more exacting and sensitive to intensive land exploitation [39]). Nevertheless, the association of contiguous altered patches may become a limiting factor for dispersal when there is a lack of continuity in suitable microhabitats among adjacent cells: this condition is able to shape the species distribution through the M-factor. The above interpretation is consistent with the analysis of the variation partitioning results (Figure 3) and the response curves (Figure 4), which show that: i) alteration of the pixel does not substantially affect suitability (no pure effect were found in both species) and the apparent trend exhibited in the single response graph of *P* *. tiliguerta* is probably due to a correlation with PC-fine; ii) altered context (PC-fine) heavily penalizes *P* *. tiliguerta* , whereas it is partly tolerated by *P* *. siculus* . This is what we might expect considering the different characteristics of the two species. In fact, the Italian wall lizard: (i) is more synanthropic than the congeneric Tyrrhenian wall lizard [39,42]; (ii) is more effective in thermoregulation (its body temperature shows little variation when the environmental temperature changes [42]), so it stands up well to the warm conditions typical of the anthropic habitats; (iii) is a better performer in locomotor endurance tests [70]; (iv) is highly effective in colonizing anthropic habitats, as long as parks and garden are available [38]. All these characteristics lead us to claim that *P* *. siculus* should have better dispersal ability in altered landscapes, and that isolation should be a less effective obstacle for this species, at least up to a certain threshold.

From the conservation point of view, our case study allows drawing some important guidelines, which may be the starting point for management actions and/or for further investigation. Firstly, the endemic species would be seriously threatened by habitat alteration rising; the same would not be true for the introduced as well as long time naturalized species. So if other natural areas will be lost, we might assist to a regression of the endemic lizard, without a meaningful change in the distribution of the introduced *P* *. siculus* . Secondly, the main cause of concern at current state is represented by habitat isolation, measured at one kilometre scale; so it would be worthwhile to focus any actions primarily to this scale in order to limit the rise of this parameter value. The importance of establishing the scale and the aim of a conservation plan does not need further discussion [15,16].

Generalizing our findings, the proposed approach, which aimed to link the spatial distribution of a species to the anthropogenic alteration of the patch context, has proved to work well, giving reasonable and ecologically interpretable results. It combines the advantages of SDM and of ITMC. The former allows using available data on species distribution, not necessarily taken for conservation aims [71], so reducing the field work effort and the related costs. Furthermore it allows simultaneously analysing larger portion of the distribution area of a species than traditional approaches, which typically use few subsamples of it [9,13,15,23]. The latter (ITMC), being a general method to compare explicit hypotheses and weigh quantifiable variable importance [32,33], may be easily applied to assess the scale at which human disturbance produces the strongest effects. The same approach can work well even with other measurement of human disturbance (for instance: pollutant concentration, invasive species presence, pesticides, road network extension): it is sufficient to translate human disturbance into variables quantifiable at different scales and then compare the models built under the null and the alternative hypotheses.

Finally, we underline that a critical step in applying our procedure is the choice of the “right” investigating scale (i.e., the resolution of the environmental and occurrence data used in developing the SDM). This choice is fundamental in order to correctly interpret patch context effect: using the same order of magnitude of the species dispersal ability may be a good idea [67], because it allows disentangling the effects of habitat loss from those of fragmentation/isolation at the species point of view [15].

## Supporting Information

Figure S1 Map of the occurrence points and the road network. (DOC)

Table S1 Spatial Correlations among topographic and climatic variables. (DOC)

Table S2 Land use conversion table. (DOC)

Table S3 Spatial correlations among the final candidate variables. (DOC)
