# Supplementary material for: Assessing the Spatial Scale Effect of Anthropogenic Factors on Species Distribution
Source: PLoS One. 2013 Jun 18;8(6):e67573. doi: 10.1371/journal.pone.0067573 (PMC3688972; doi:10.1371/journal.pone.0067573)
Supplement: Figure S1 — (DOC) [file pone.0067573.s001.doc]

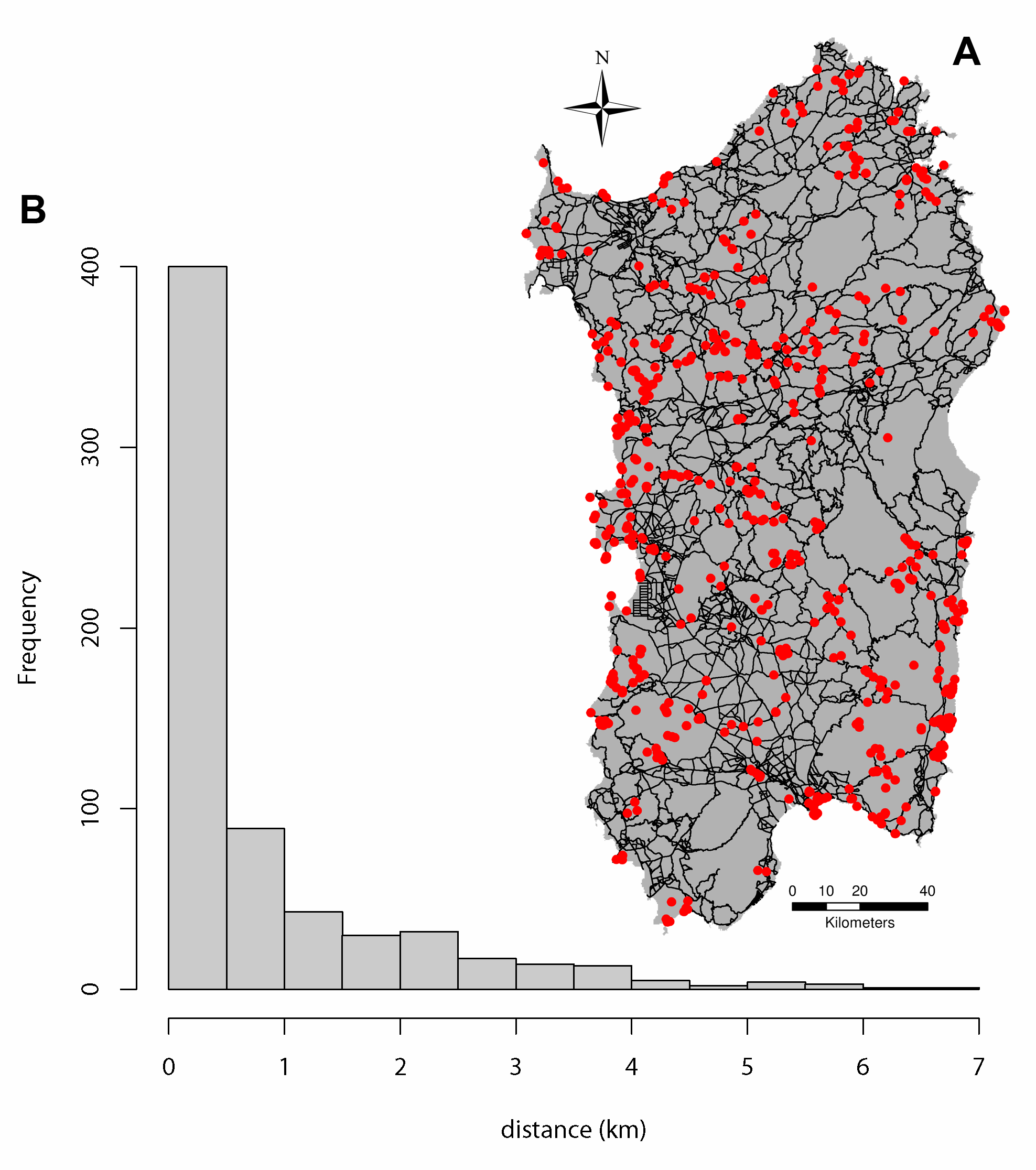
**Figure S4**. *A*. Map showing the occurrence points of the study species (red points) and the road network of Sardinia (black lines). We used roads to select background points for model building. See text for details. *B.* Histogram of the distances from each occurrence point to the nearest road.
