# Supplementary material for: Assessing the Spatial Scale Effect of Anthropogenic Factors on Species Distribution
Source: PLoS One. 2013 Jun 18;8(6):e67573. doi: 10.1371/journal.pone.0067573 (PMC3688972; doi:10.1371/journal.pone.0067573)
Supplement: Table S1 — (DOC) [file pone.0067573.s002.doc]

|  | **slope** | **PSR** | bio1 | bio2 | bio3 | bio4 | bio5 | bio6 | bio7 | bio8 | bio9 | bio10 | bio11 | bio12 | bio13 | bio14 | bio15 | bio16 | bio17 | bio18 | bio19 |
| --- | --- | --- | --- | --- | --- | --- | --- | --- | --- | --- | --- | --- | --- | --- | --- | --- | --- | --- | --- | --- | --- |
| **altitude** | 0.56 | 0.39 | -0.94 | -0.21 | -0.64 | 0.86 | -0.62 | -0.88 | 0.47 | -0.86 | -0.86 | -0.87 | -0.94 | 0.77 | 0.65 | 0.85 | -0.38 | 0.67 | 0.85 | 0.90 | 0.76 |
| **slope** |  | -0.07 | -0.50 | -0.30 | -0.47 | 0.36 | -0.46 | -0.38 | 0.05 | -0.45 | -0.49 | -0.49 | -0.47 | 0.34 | 0.25 | 0.48 | -0.32 | 0.27 | 0.45 | 0.52 | 0.32 |
| **PSR** |  |  | -0.38 | 0.02 | -0.18 | 0.40 | -0.20 | -0.41 | 0.30 | -0.36 | -0.34 | -0.35 | -0.39 | 0.38 | 0.35 | 0.32 | -0.06 | 0.35 | 0.34 | 0.33 | 0.38 |
| bio1 |  |  |  | 0.22 | 0.63 | -0.83 | 0.69 | 0.92 | -0.47 | 0.88 | 0.93 | 0.95 | 0.98 | -0.80 | -0.66 | -0.89 | 0.40 | -0.70 | -0.90 | -0.93 | -0.78 |
| bio2 |  |  |  |  | 0.84 | 0.11 | 0.75 | -0.05 | 0.64 | 0.01 | 0.38 | 0.37 | 0.13 | -0.13 | -0.13 | -0.40 | 0.43 | -0.12 | -0.42 | -0.44 | -0.03 |
| bio3 |  |  |  |  |  | -0.39 | 0.83 | 0.42 | 0.19 | 0.42 | 0.69 | 0.69 | 0.59 | -0.46 | -0.39 | -0.77 | 0.58 | -0.40 | -0.78 | -0.80 | -0.38 |
| bio4 |  |  |  |  |  |  | -0.30 | -0.93 | 0.78 | -0.77 | -0.65 | -0.66 | -0.89 | 0.68 | 0.58 | 0.73 | -0.32 | 0.59 | 0.74 | 0.74 | 0.69 |
| bio5 |  |  |  |  |  |  |  | 0.47 | 0.21 | 0.55 | 0.86 | 0.83 | 0.60 | -0.57 | -0.51 | -0.72 | 0.41 | -0.51 | -0.75 | -0.78 | -0.50 |
| bio6 |  |  |  |  |  |  |  |  | -0.70 | 0.86 | 0.80 | 0.81 | 0.95 | -0.73 | -0.60 | -0.78 | 0.36 | -0.63 | -0.79 | -0.80 | -0.74 |
| bio7 |  |  |  |  |  |  |  |  |  | -0.52 | -0.24 | -0.27 | -0.56 | 0.38 | 0.31 | 0.29 | -0.03 | 0.32 | 0.30 | 0.27 | 0.43 |
| bio8 |  |  |  |  |  |  |  |  |  |  | 0.83 | 0.82 | 0.87 | -0.77 | -0.60 | -0.75 | 0.25 | -0.66 | -0.73 | -0.77 | -0.81 |
| bio9 |  |  |  |  |  |  |  |  |  |  |  | 0.98 | 0.88 | -0.79 | -0.68 | -0.84 | 0.34 | -0.70 | -0.86 | -0.90 | -0.75 |
| bio10 |  |  |  |  |  |  |  |  |  |  |  |  | 0.91 | -0.79 | -0.69 | -0.87 | 0.37 | -0.71 | -0.89 | -0.92 | -0.76 |
| bio11 |  |  |  |  |  |  |  |  |  |  |  |  |  | -0.80 | -0.68 | -0.88 | 0.37 | -0.71 | -0.90 | -0.91 | -0.80 |
| bio12 |  |  |  |  |  |  |  |  |  |  |  |  |  |  | 0.94 | 0.67 | 0.12 | 0.97 | 0.79 | 0.78 | 0.98 |
| bio13 |  |  |  |  |  |  |  |  |  |  |  |  |  |  |  | 0.53 | 0.29 | 0.98 | 0.69 | 0.67 | 0.92 |
| bio14 |  |  |  |  |  |  |  |  |  |  |  |  |  |  |  |  | -0.56 | 0.56 | 0.94 | 0.95 | 0.65 |
| bio15 |  |  |  |  |  |  |  |  |  |  |  |  |  |  |  |  |  | 0.26 | -0.41 | -0.43 | 0.14 |
| bio16 |  |  |  |  |  |  |  |  |  |  |  |  |  |  |  |  |  |  | 0.72 | 0.70 | 0.96 |
| bio17 |  |  |  |  |  |  |  |  |  |  |  |  |  |  |  |  |  |  |  | 0.98 | 0.75 |
| bio18 |  |  |  |  |  |  |  |  |  |  |  |  |  |  |  |  |  |  |  |  | 0.75 |

**Table S1**. Spearman correlation coefficients among the three variables used (in bold) and the 19 bioclimatic variables of worldclim (Hijmans et al., 2005; [www.worldclim.org](http://www.worldclim.org/)). Variables abbreviations are as follows: PSR = Potential Solar Radiation; bio1 = Annual Mean Temperature, bio2 = Mean Diurnal Range; bio3 = Isothermality ; bio4 = Temperature Seasonality; bio5 = Max Temperature of Warmest Month; bio6 = Min Temperature of Coldest Month; bio7 = Temperature Annual Range; bio8 = Mean Temperature of Wettest Quarter; bio9 = Mean Temperature of Driest Quarter; bio10 = Mean Temperature of Warmest Quarter; bio11 = Mean Temperature of Coldest Quarter; bio12 = Annual Precipitation; bio13 = Precipitation of Wettest Month; bio14 = Precipitation of Driest Month; bio15 = Precipitation Seasonality; bio16 = Precipitation of Wettest Quarter; bio17 = Precipitation of Driest Quarter; bio18 = Precipitation of Warmest Quarter; bio19 = Precipitation of Coldest Quarter. For legend details see Hijmans et al. (2005).

Hijmans, R.J., Cameron, S.E., Parra, J.L., Jones, P.G., Jarvis, A. (2005). Very high resolution interpolated climate surfaces for global land areas. Int. Journal of Climatology, 25: 1965-1978.
