# Supplementary material for: Assessing the Spatial Scale Effect of Anthropogenic Factors on Species Distribution
Source: PLoS One. 2013 Jun 18;8(6):e67573. doi: 10.1371/journal.pone.0067573 (PMC3688972; doi:10.1371/journal.pone.0067573)
Supplement: Table S2 — (DOC) [file pone.0067573.s003.doc]

| **Land use description** | **Cover (%)** | **Altered** |
| --- | --- | --- |
| Deciduous forests | 14.60% | 0 |
| Mediterranean scrubs | 14.17% | 0 |
| Crops without irrigation systems | 10.42% | 1 |
| Garrigue | 9.13% | 0 |
| Cropland | 8.55% | 1 |
| Artificial grass | 6.84% | 0 |
| Natural pastures | 6.01% | 0 |
| Areas of natural re-colonization | 3.86% | 0 |
| Corks | 3.34% | 0 |
| Mixed temporal and permanent crops | 2.41% | 1 |
| Agroforestry areas | 2.39% | 0 |
| Areas of artificial re-colonization | 2.03% | 0 |
| Olive groves | 2.02% | 0 |
| Complex crops systems | 1.75% | 1 |
| Coniferous forest | 1.53% | 0 |
| Crops with natural area inside | 1.21% | 1 |
| Vineyards | 1.02% | 1 |

**Table S2**. Land use conversion table used to build variable “alteration”. For each category of land use we reported the relative cover (as percentage) and the assigned binary score (1 = altered, 0= not altered) based on the definition given in main text. We omitted categories whose cover is less than 1%.
