# Supplementary material for: Assessing the Spatial Scale Effect of Anthropogenic Factors on Species Distribution
Source: PLoS One. 2013 Jun 18;8(6):e67573. doi: 10.1371/journal.pone.0067573 (PMC3688972; doi:10.1371/journal.pone.0067573)
Supplement: Table S3 — (DOC) [file pone.0067573.s004.doc]

|  | PC-150 | PC-500 | **PC-1000** | PC-5000 | PC-10000 | **PC-15000** | PC-20000 | **Altitude** | **Slope** | **PSR** | **bio2** | **bio7** | **bio13** | **bio15** |
| --- | --- | --- | --- | --- | --- | --- | --- | --- | --- | --- | --- | --- | --- | --- |
| **Alteration** | **0.85** | **0.75** | 0.68 | 0.62 | 0.53 | 0.47 | 0.42 | 0.39 | -0.38 | -0.50 | -0.03 | 0.28 | 0.03 | -0.24 |
| PC-150 |  | **0.91** | **0.83** | **0.76** | 0.66 | 0.58 | 0.52 | 0.48 | -0.47 | -0.59 | -0.05 | 0.34 | 0.02 | -0.30 |
| PC-500 |  |  | **0.94** | **0.87** | **0.75** | 0.67 | 0.60 | 0.55 | -0.54 | -0.63 | -0.06 | 0.39 | 0.02 | -0.33 |
| **PC-1000** |  |  |  | **0.95** | **0.84** | **0.74** | 0.66 | 0.60 | -0.58 | -0.65 | -0.06 | 0.43 | 0.03 | -0.36 |
| PC-5000 |  |  |  |  | **0.92** | **0.82** | **0.73** | 0.66 | -0.60 | -0.64 | -0.07 | 0.46 | 0.03 | -0.37 |
| PC-10000 |  |  |  |  |  | **0.93** | **0.84** | **0.77** | -0.60 | -0.60 | -0.07 | 0.50 | 0.06 | -0.34 |
| **PC-15000** |  |  |  |  |  |  | **0.95** | **0.89** | -0.57 | -0.56 | -0.06 | 0.54 | 0.10 | -0.28 |
| PC-20000 |  |  |  |  |  |  |  | **0.97** | -0.51 | -0.50 | -0.04 | 0.57 | 0.15 | -0.21 |
| **Altitude** |  |  |  |  |  |  |  |  | -0.46 | -0.46 | -0.02 | 0.61 | 0.20 | -0.16 |
| **Slope** |  |  |  |  |  |  |  |  |  | 0.57 | 0.39 | -0.22 | 0.47 | 0.65 |
| **PSR** |  |  |  |  |  |  |  |  |  |  | -0.07 | -0.31 | 0.05 | 0.25 |
| **bio2** |  |  |  |  |  |  |  |  |  |  |  | 0.01 | 0.31 | 0.35 |
| **bio7** |  |  |  |  |  |  |  |  |  |  |  |  | 0.64 | -0.14 |
| **bio13** |  |  |  |  |  |  |  |  |  |  |  |  |  | 0.30 |

**Table S3**. Spearman correlation coefficients among the candidate variables used to build the spatial distribution models. PSR = “Potential Solar Radiation”; bio2 = Mean Diurnal Range; bio7 = Temperature Annual Range; bio13 = Precipitation of Wettest Month; bio15 = Precipitation Seasonality; PC-150, PC-500, PC-1000 etc. stand for “Patch Context” followed by the distance in meters used to compute the context alteration. Bolded names indicate chosen variables; bolded numbers are the values of correlation coefficients that exceed the 0.70 threshold.
